# Incidence of Visuospatial Neglect in Acute Stroke: Assessment and Stroke Characteristics in an Unselected 1-Year Cohort

**DOI:** 10.1161/STROKEAHA.124.048907

**Published:** 2025-04-07

**Authors:** Dario Cazzoli, Brigitte C. Kaufmann, Henrik Rühe, Nora Geiser, Thomas Nyffeler

**Affiliations:** Neurocenter, Lucerne Cantonal Hospital, University Teaching and Research Hospital (D.C., B.C.K., H.R., N.G., T.N.), University of Lucerne, Switzerland.; Faculty of Behavioural Sciences and Psychology (D.C.), University of Lucerne, Switzerland.; Faculty of Health Sciences and Medicine (T.N.), University of Lucerne, Switzerland.; Department of Psychology (D.C.), University of Bern, Switzerland.; ARTORG Center for Biomedical Engineering Research (D.C., N.G., T.N.), University of Bern, Switzerland.; Graduate School for Health Sciences (N.G.), University of Bern, Switzerland.; Department of Neurology, Inselspital, Bern University Hospital (T.N.), University of Bern, Switzerland.

**Keywords:** attention, cognitive dysfunction, epidemiology, neurological rehabilitation, symptom assessment

## Abstract

**BACKGROUND:**

The true incidence of visuospatial neglect, impaired attention toward contralesional space, remains unclear. Common variability sources are sensitivity differences of conventional assessments and the exclusion of patients with language, motor, and other cognitive impairments. We aimed to determine the incidence of visuospatial neglect in an unselected cohort of patients with acute stroke using video oculography during free visual exploration, a newly established assessment overcoming the aforementioned biases.

**METHODS:**

Single-center, prospective, observational cohort study. We screened every patient admitted to a representative Swiss stroke center over 1 year (N=626). Two hundred eighty-five patients were eligible (first-ever stroke within 72 hours), and 221 were included. The incidence of visuospatial neglect was determined with conventional paper-pencil assessments and video oculography during free visual exploration. Demographic, risk, and stroke-related factors, as well as stroke localization, were also considered. Feasibility and ability to detect visuospatial neglect of the assessments were evaluated.

**RESULTS:**

The overall incidence of visuospatial neglect was ≈38%: widely varying location-specifically: ≈61% and ≈22% for stroke in the right and left cerebral hemispheres, respectively, and ≈14% to ≈37% for some less commonly affected infratentorial areas or multifocal stroke. In hemispheric stroke, visuospatial neglect was most common when the middle (≈64% right and ≈21% left) and posterior (≈53% right and ≈25% left) cerebral artery territories were affected. Neglect patients had higher National Institutes of Health Stroke Scale scores, more commonly atrial fibrillation and thrombectomy, and less commonly an undetermined stroke cause. They were older, with ≈4% yearly increase in the odds of having visuospatial neglect. Video oculography during free visual exploration was administrable and detected visuospatial neglect more often than conventional paper-pencil assessments.

**CONCLUSIONS:**

The incidence of visuospatial neglect in an unselected cohort, using a highly sensitive assessment, is considerably higher than previously assumed and can also occur after less typically localized strokes. These results can enhance the awareness of visuospatial neglect in the acute setting, potentially facilitating earlier identification and therapy of this disabling disorder.

Visuospatial neglect, defined as the inability to attend or respond toward the contralesional space, is a negative and independent predictor of poor outcome after stroke: patients with visuospatial neglect have a slower rehabilitation progress, increased hospitalization length, and lower likelihood of being discharged home, all also resulting in higher costs.^1^ An early identification and therapy of visuospatial neglect are, therefore, of crucial importance. However, the incidence of visuospatial neglect after acute stroke is still a matter of debate. When considering newer studies (ie, after 2000), a recent systematic review^2^ indicated an average incidence of 38% (range, 19%–47%) for acute right-hemispheric stroke and 17% (range, 2%–24%) for acute left-hemispheric stroke. Interestingly, these figures increased for the subacute phase (46% for right-hemispheric subacute stroke and 19% for left-hemispheric subacute stroke). This counterintuitive increase, which is contrary to the natural course of remission over time, together with the large variability in incidence, suggests that visuospatial neglect is not reliably detected in acute stroke and is, therefore, underdiagnosed.

One of the sources of this variability is the considerable differences in sensitivity of the applied assessment methods.^3^ For instance, the diagnostic sensitivity of the item Extinction and Inattention (formerly Neglect) of the National Institutes of Health Stroke Scale (NIHSS) is poor.^4^ Also, paper-pencil tests (eg, canceling targets among distractors or bisecting lines on a paper sheet), which are often applied instead, have a low sensitivity^2,5^ and are often not feasible due to motor, language, and cognitive impairments other than neglect. Finally, the systematic, ecological observation of spontaneous behavior during the activities of daily living, one of the most sensitive assessment methods,^2,5^ is often not feasible in a stroke unit setting because of practical (eg, patient condition) and time constraints.^6^

Recently, video oculography during free visual exploration (FVE) was established as a new method for diagnosing visuospatial neglect,^7–9^ being included in a current shortlist of methods with the best available evidence of both validity and reliability.^10^ FVE overcomes most of the abovementioned issues because it (1) has high sensitivity and specificity in detecting visuospatial neglect, significantly higher than conventionally used paper-pencil tests and their combination,^7^ even in subtle forms of visuospatial neglect^9^; (2) significantly correlates with observational measures of visuospatial neglect during the activities of daily living (Catherine Bergego Scale^5^), thus providing an ecologically valid and relevant assessment^7^; (3) requires minimal, nonverbal instructions and does not require manual motor output, making it suitable also for the assessment of patients with aphasia and apraxia^8^; and (4) has a short administration time (<10 minutes overall), making it suitable for the assessment in the acute stroke setting.

In this single-center, prospective, observational cohort study, we aimed to determine the incidence of visuospatial neglect in acute stroke using conventional paper-pencil tests and the newly established FVE. To this end, over a period of 1 year, we conducted a prospective examination of an unselected cohort, that is, each patient presenting with a first-ever stroke within 72 hours of symptom onset who was admitted to a stroke center. In addition, we considered associated demographic, risk, and stroke-related factors.

## Methods

### Materials and Data Availability

Our ethics approval does not permit public archiving of the data. Based on the Swiss Human Research Act, readers seeking access to data and study materials must contact the corresponding author and complete a formal data-sharing agreement.

### Study Population

This single-center, prospective, observational cohort study was designed to investigate the incidence of visuospatial neglect attributable to acute first-ever stroke. We considered the collective of patients treated at the Stroke Center of the Lucerne Cantonal Hospital, Switzerland, as a representative and suitable model for this purpose. The Stroke Center of the Lucerne Cantonal Hospital is the only one certified in central Switzerland and covers a large, both urban and rural, catchment area.

All patients admitted to the Stroke Center of the Lucerne Cantonal Hospital between July 1, 2022, and June 30, 2023, were screened. Inclusion criteria were (1) first-ever stroke, as confirmed by computed tomography or magnetic resonance imaging performed during clinical workup; (2) within a time window of 72 hours after symptom onset; and (3) aged ≥18 years. Exclusion criteria were the inability to provide informed consent, even with nonverbal instructions or the support of relatives. We purposefully did not introduce any other inclusion/exclusion criteria (eg, concerning stroke type or localization) to keep the study population as representative as possible. The STROBE guideline (Strengthening the Reporting of Observational Studies in Epidemiology) was followed (Supplemental Material).

**Figure 1.**
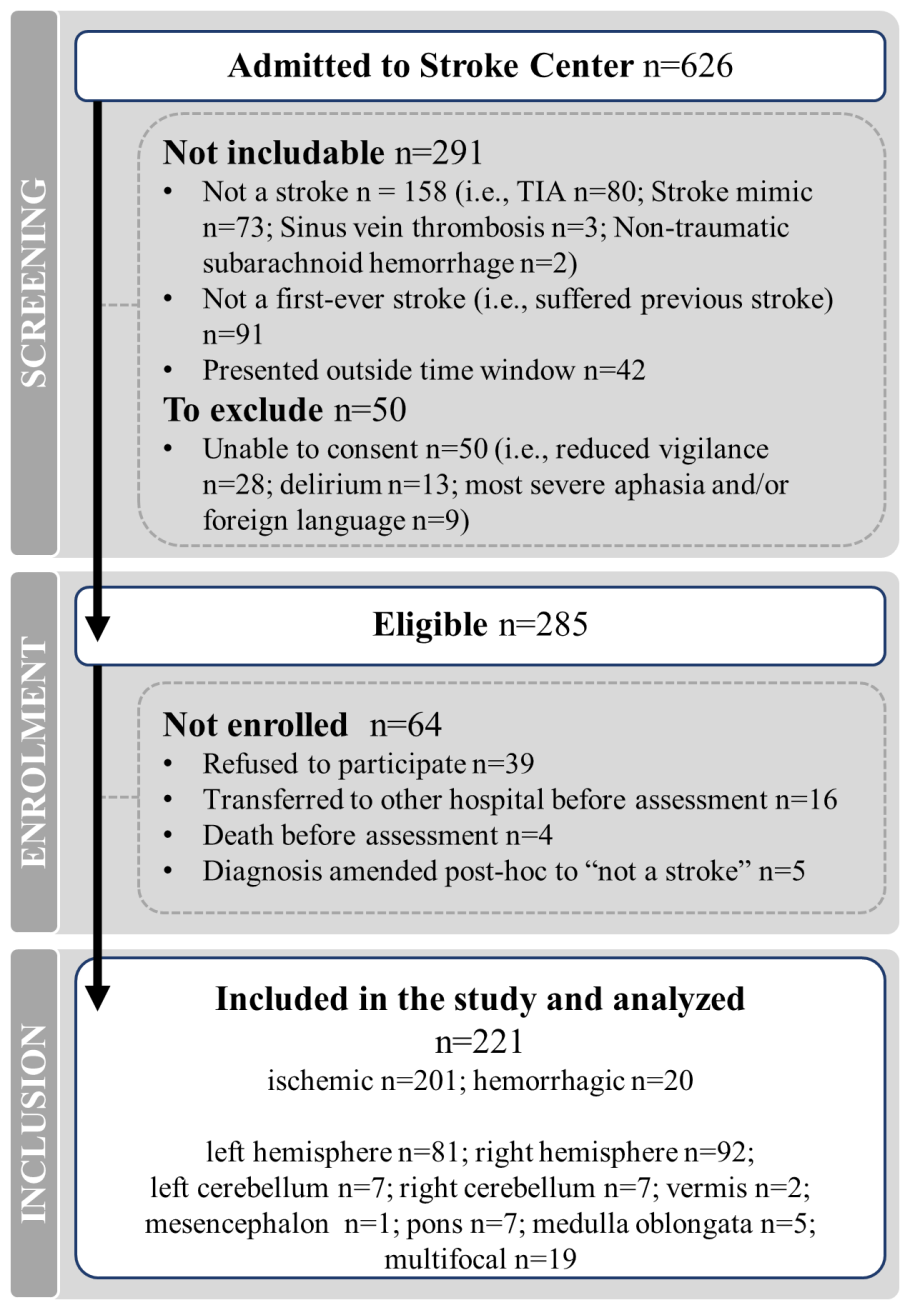
**STROBE (Strengthening the Reporting of Observational Studies in Epidemiology) inclusion flowchart.** Six hundred twenty-six patients were admitted to the Stroke Center of the Lucerne Cantonal Hospital, Switzerland, between July 1, 2022, and June 30, 2023, and were screened. Two hundred eighty-five patients were eligible; 221 could be included in the study, and their data were analyzed. TIA indicates transient ischaemic attack.

We screened a total of 626 patients. After applying inclusion/exclusion criteria, 285 patients were eligible for participation. Thereof, 221 could be enrolled, and their data are included in the final analyses (Figure 1).

The study was approved by the local ethics committee (ID 2022-01079) and performed according to the latest Declaration of Helsinki. All patients gave written informed consent before participation.

### Assessment of Visuospatial Neglect

Visuospatial neglect was evaluated with 3 assessments (FVE and 2 paper-pencil tests), administered in random order in one testing session.

FVE measured the spatial distribution of visual fixations on the horizontal plane, as the primary assessment. Patients freely explored 12 images of natural scenes or urban public places in random order (6 original and 6 mirrored versions),^11^ presented on a laptop screen for 7 seconds each. A central, black fixation cross on a gray background (1 second, before each image) enforced a common exploration starting point. Video oculography was performed with a remote, infrared- and video-based eye-tracking system (EyeLink Portable Duo, SR Research, Ottawa, ON, Canada). The system was placed on an overbed table, allowing bedside testing, with the patients’ bed backrest placed in a vertical position. The screen was placed at 59 cm from the patients’ eyes, resulting in a visual angle of 28°×21°, with the screen center and patients’ midsagittal plane aligned. System calibration and validation were performed with 3×3 points grids. FVE lasted <10 minutes. All fixations with a duration of 100 to 2000 ms^12^ were included in the data analyses (mean number of fixations excluded=10.821%; SD, 11.346%). The mean gaze position (the mean horizontal position of all fixations in ° of visual angle from the screen center) was computed. A schematic of the free visual exploration paradigm and further details of its administration are provided in the Supplemental Methods (Figure S1).

To compare the performance of FVE in detecting visuospatial neglect with the one of commonly used paper-pencil tests, we administered the Bells test^13^ and a line bisection test, in which patients were asked to mark their perceived midpoint of 15 horizontal lines of varying length (6, 12, and 18 cm, 5 lines per length, in mixed order and equally spaced) and horizontal placement (far and near left and right, and central).^14^ The paper-pencil tests were printed on a horizontally oriented A4 sheet of paper and presented on an empty overbed table, with the center of the sheet of paper aligned with the patients’ midsagittal plane and fixed with tape to prevent movement during test performance. For the Bells test, we computed the Center of Cancellation.^15^ For the line bisection test, we calculated the deviation between the actual and marked midlines as a percentage of the true half of the line according to the formula proposed by Schenkenberg et al^16^; a mean of the percentage deviations of all lines was then calculated.

Pathological scores were defined as mean gaze position ≥1.333° and ≤−1.359°,^7,8^ Center of Cancellation ≥0.081 and ≤−0.086,^17^ and mean percent deviation ≥7.07% and ≤−5.34%,^14^ for left- and right-sided visuospatial neglect, respectively.

### Statistical Analysis

We defined the presence of visuospatial neglect as a pathological score in at least 1 of the 3 assessments (ie, FVE, the Bells test, and the line bisection test). We computed the incidence of visuospatial neglect (specifically, the incidence proportion, that is, the number of new cases of visuospatial neglect divided by the number of patients in the at-risk population of an unselected cohort presenting with a first-ever stroke within 72 hours of symptom onset and admitted to a representative stroke center over a 1-year period), including 95% CI. We then compared the 2 groups of patients with and without visuospatial neglect with respect to demographic characteristics (age, sex, and handedness), NIHSS score, treatment with thrombolysis or thrombectomy, stroke risk factors (hypertension, diabetes, hyperlipidemia, smoking or history of smoking, excessive alcohol consumption, atrial fibrillation, and coronary heart disease), and stroke cause according to the TOAST (Trial of ORG 10172 in Acute Stroke Treatment) classification,^18^ including odds ratios (ORs) and 95% CIs thereof.

We then considered the incidence (again, specifically the incidence proportion) of visuospatial neglect according to first-ever stroke localization in macroscopic brain segments (left cerebral hemisphere, right cerebral hemisphere, left cerebellar hemisphere, right cerebellar hemisphere, cerebellar vermis, mesencephalon, pons, medulla oblongata, and multifocal, ie, >1 of these segments), as assessed with computed tomography (Siemens Somatom Force Dual Source or X-Ceed) or magnetic resonance imaging (Siemens Magnetom Aera 1.5T, Vida fit 3.0T, or Philips Achieva 3.0T) included in the clinical stroke workup. Because first-ever stroke occurred far more commonly within the cerebral hemispheres, we conducted additional analyses considering visuospatial neglect incidence according to vascularization territories (anterior cerebral artery, middle cerebral artery [MCA], and posterior cerebral artery [PCA]) for the left and right cerebral hemispheres, respectively. We computed the incidence of visuospatial neglect in the groups of patients with first-ever stroke in each brain segment and cerebral vascularization territory.

We compared the groups with independent *t* tests for continuous variables (Mann-Whitney *U* tests for nonnormally distributed variables, ie, the significant Shapiro-Wilk test) and with χ^2^ tests for dichotomous variables. For first-ever stroke brain segments and vascularization territory groups with >10 patients, we assessed differences in the likelihood of presenting with visuospatial neglect or not with χ^2^ tests.

Finally, we assessed the feasibility of the 3 assessments (FVE, the Bells test, and the line bisection test) and their ability to detect visuospatial neglect. For these analyses, we focused on the largest cohort of our patient sample, that is, patients with first-ever stroke with lesions in the left or the right cerebral hemisphere. Feasibility was determined by whether the assessments could be successfully administered and completed by the patients. We computed the numbers (and percentages) of patients for whom each assessment was feasible and capable of detecting visuospatial neglect, analyzing the data separately for patients with left- and right-hemispheric cerebral first-ever strokes. These values were compared using McNemar tests for dichotomous variables.

All *P* values were 2-tailed; statistical significance was set at *P*<0.05. In cases where multiple testing was performed (ie, when comparing the feasibility and ability to detect visuospatial neglect of the respective assessments and combinations thereof), *P* values were corrected by controlling for the false discovery rate using the Benjamini-Hochberg procedure.^19^ The respective *P* values are reported as *P*_corr_.

The shared first authors had full access to all data and take responsibility for its integrity and analysis.

## Results

### Visuospatial Neglect Incidence at the Study Population Level and Effects of Demographic and Clinical Characteristics

Among the 221 patients with first-ever stroke included in the present study, 84 had visuospatial neglect (defined as a pathological score in at least 1 of the 3 assessments, ie, FVE, the Bells test, and the line bisection test), yielding an overall incidence of 38.01% (95% CI, 31.60%–44.4%).

Demographic and clinical characteristics of the population, split according to the presence or absence of visuospatial neglect, are presented in the Table. In addition, the clinical data concerning a brief screening of general cognitive abilities (Montreal Cognitive Assessment^20^) were also retrospectively searched and available (ie, when practical and time constraints allowed in the acute stroke setting) for 171 of the 221 included patients with first-ever stroke (77.38%); the respective results are reported in the Supplemental Results.

**Table.**
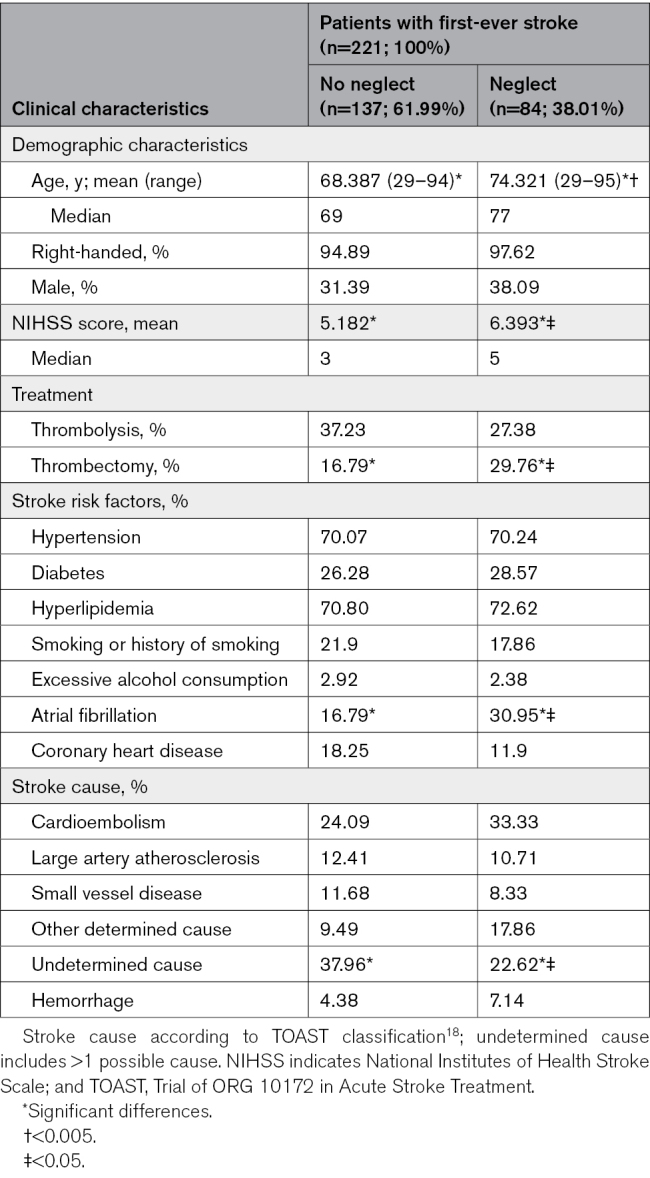
Demographic and Clinical Characteristics, Treatment With Thrombolysis or Thrombectomy, Stroke Risk Factors, and Cause Among Patients With First-Ever Stroke With Versus Without Visuospatial Neglect

**Figure 2.**
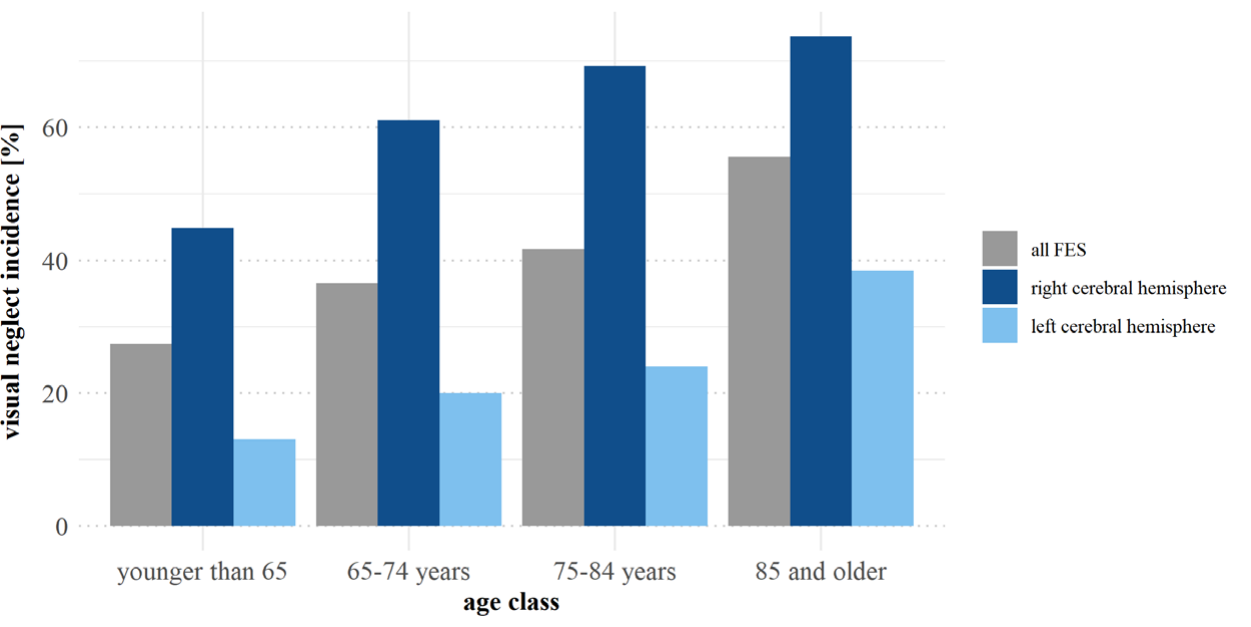
**Neglect incidence according to increasing age classes.** The bar plots represent the data for the whole sample of patients with first-ever stroke (FES; gray) and for patients with first-ever stroke within the right (dark blue) and left (light blue) cerebral hemispheres.

Patients with first-ever stroke with visuospatial neglect were significantly older than their peers without visuospatial neglect (*P*=0.002). An evaluation of age classes showed a constant increase in visuospatial neglect incidence with growing age (Figure 2). An additional logistic regression model showed that increasing age is a significant predictor of the incidence of visuospatial neglect (*P*=0.003; OR, 1.039), indicating that every additional increase in 1 year in age is associated with a 3.9% increase in the odds of having visuospatial neglect when suffering from first-ever stroke, even when controlling for other significant factors (NIHSS score, atrial fibrillation, and thrombectomy; see the following). There was no significant difference in visuospatial neglect incidence concerning sex (*P*=0.307; OR, 1.345 [95% CI, 0.761–2.377]) or handedness (*P*=0.319; OR, 2.208 [95% CI, 0.448–10.887]).

Patients with first-ever stroke with visuospatial neglect presented with a significantly higher NIHSS score than their peers without visuospatial neglect (*P*=0.023); a reanalysis of the NIHSS data excluding the Extinction and Inattention (formerly Neglect) item revealed a tendency for patients with first-ever stroke with visuospatial neglect to have higher NIHSS scores than their peers without visuospatial neglect (for details, see the Supplemental Results). Moreover, patients with first-ever stroke with visuospatial neglect were significantly more likely to receive thrombectomy (*P*=0.023; OR, 2.1 [95% CI, 1.099–4.014]) but not thrombolysis (*P*=0.132; OR, 0.636 [95% CI, 0.352–1.149]).

**Figure 3.**
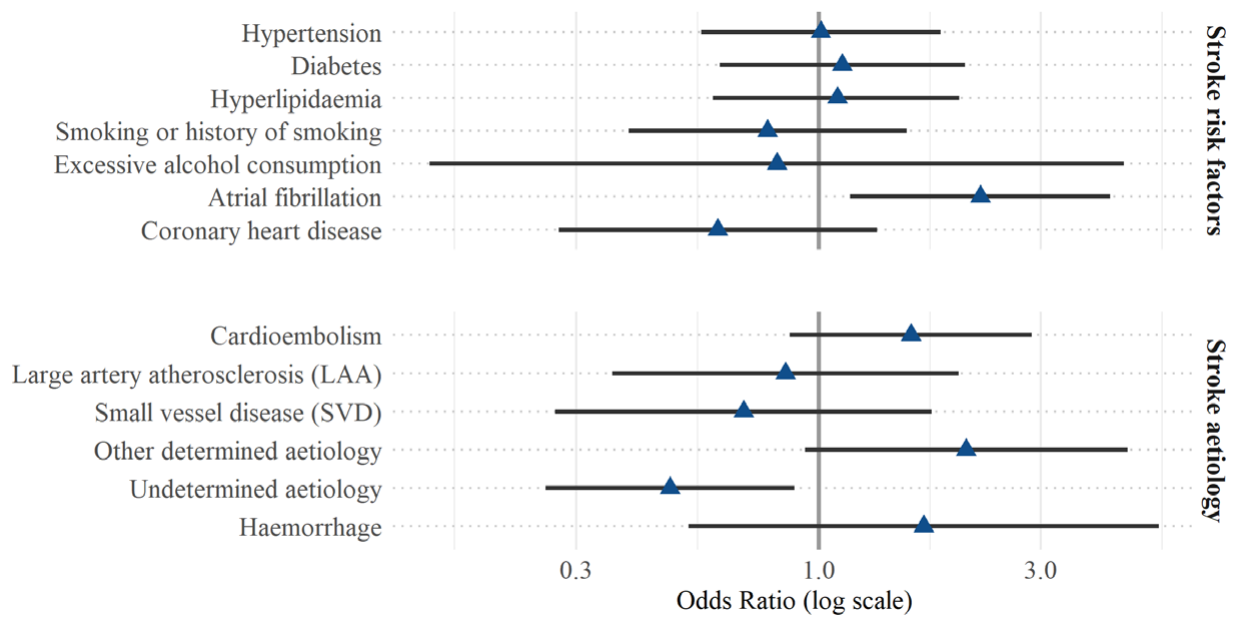
**Odds ratios for stroke risk factors and stroke etiology.** Forest plot showing odds ratios (blue triangles, on the log scale) and respective 95% CIs (black whiskers) of presenting with visuospatial neglect after first-ever stroke according to stroke risk factors (top) and cause (lower). Stroke cause was defined according to the TOAST (Trial of ORG 10172 in Acute Stroke Treatment) classification^18^; thereby, an undetermined cause includes >1 possible cause.

ORs and respective 95% CIs concerning stroke risk factors and causes are presented in Figure 3.

Atrial fibrillation was significantly more common in patients with first-ever stroke with visuospatial neglect than in their peers without visuospatial neglect (*P*=0.014; OR, 2.222 [95% CI, 1.167–4.231]). Other risk factors did not differ between the 2 groups (hypertension: *P*=0.979; diabetes: *P*=0.710; hyperlipidemia: *P*=0.772; coronary heart disease: *P*=0.210; excessive alcohol consumption: *P*=0.811; and smoking or history of smoking: *P*=0.469).

An undetermined cause was significantly less common in patients with first-ever stroke with visuospatial neglect than in their peers without visuospatial neglect (*P*=0.018). The occurrence of other etiologies did not differ between the 2 groups (cardioembolism: *P*=0.136; small vessel disease, SVD: *P*=0.429; other determined cause: *P*=0.069; large artery atherosclerosis: *P*=0.704; and hemorrhage: *P*=0.379).

### Visuospatial Neglect Incidence According to First-Ever Stroke Localization in Macroscopic Brain Segments

**Figure 4.**
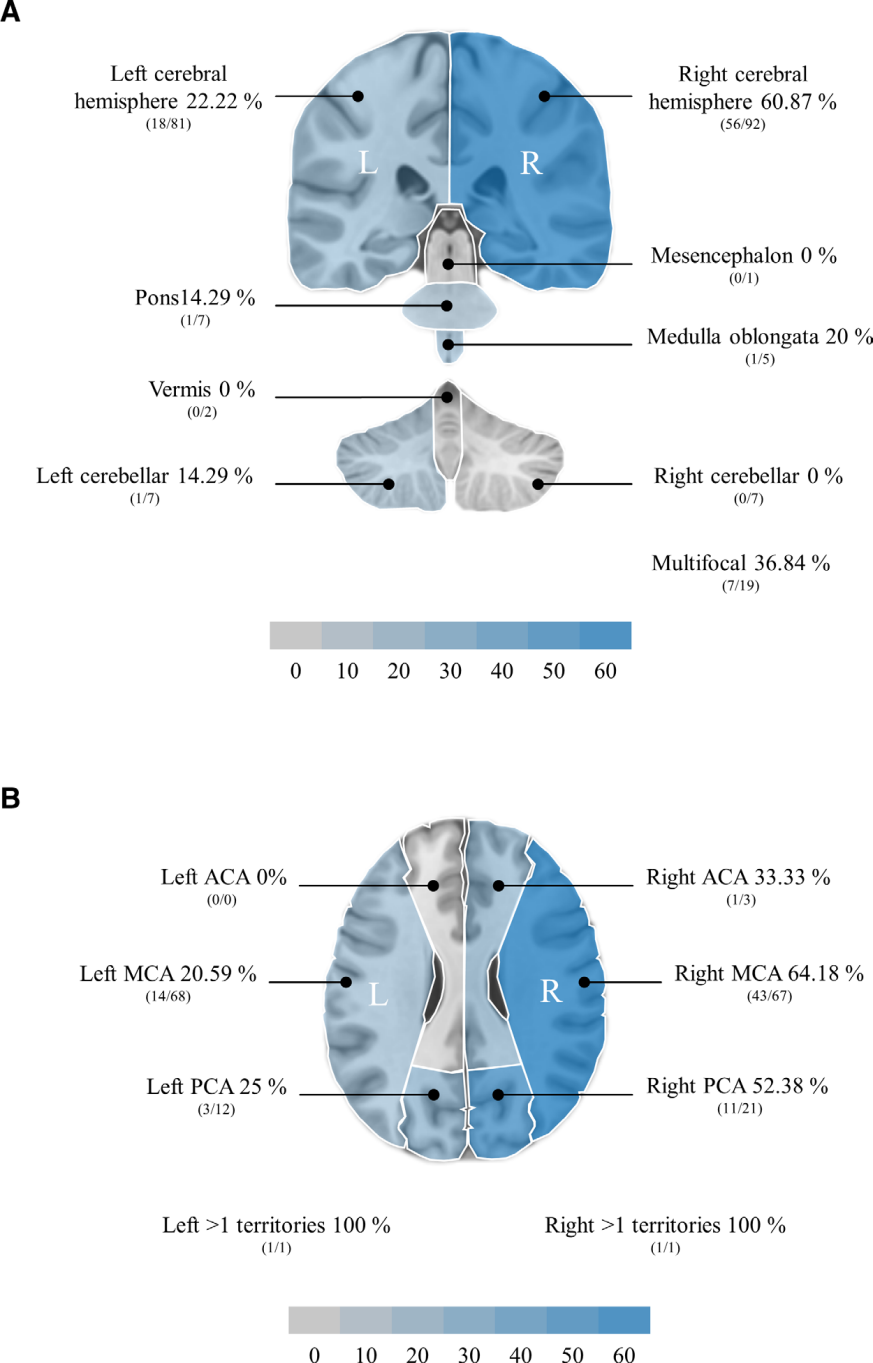
**Incidence of visuospatial neglect per brain segments and vascularization territories.** Graphical representation of the incidence of visuospatial neglect according to first-ever stroke localization in macroscopic brain segments (**A**) and according to vascularization territories within the cerebral hemispheres (**B**). Incidences are presented in percentages (also color-coded) and in absolute numbers (in brackets). ACA indicates anterior cerebral artery; MCA, middle cerebral artery; and PCA, posterior cerebral artery.

The incidence of visuospatial neglect after the first-ever stroke showed a large difference between the 2 cerebral hemispheres (Figure 4A). While 60.87% of patients with a first-ever stroke in the right cerebral hemisphere had visuospatial neglect, the proportionally smaller percentage of 22.22% of patients with a first-ever stroke in the left cerebral hemisphere had visuospatial neglect. Indeed, for patients with right-hemispheric first-ever stroke, it was significantly more likely to have visuospatial neglect than not to have one (*P*<0.001), while the reverse applied to patients with left-hemispheric first-ever stroke (*P*<0.001).

Interestingly, visuospatial neglect was also observed in patients with first-ever stroke in brain segments that were less commonly affected, such as the left cerebellum, the pons, and the medulla oblongata. The incidence of visuospatial neglect after the first-ever stroke in these locations was comparatively lower but not negligible, ranging from ≈14% to 20% (Figure 4A). Visuospatial neglect was also observable in patients with multifocal first-ever stroke, with an incidence of 36.84%, with no significant difference in likelihood (*P*=0.251).

Although first-ever stroke was rare in these brain segments, making a reliable interpretation of the data difficult, no visuospatial neglect was observed after the first-ever stroke in the right cerebellum, vermis, or mesencephalon.

### Visuospatial Neglect Incidence After First-Ever Stroke Within the Cerebral Hemispheres According to Vascularization Territories

The incidence of visuospatial neglect after the first-ever stroke in the MCA territory showed a large difference between the 2 cerebral hemispheres (Figure 4B). While 64.18% of patients with a right-hemispheric first-ever stroke in the MCA territory had visuospatial neglect, the proportionally smaller percentage of 20.59% of patients with a left-hemispheric first-ever stroke in the MCA territory had visuospatial neglect. Indeed, for patients with a right-hemispheric first-ever stroke in the MCA territory, it was significantly more likely to have visuospatial neglect than not to have one (*P*=0.020), while the reverse applied to patients with a left-hemispheric first-ever stroke in the MCA territory (*P*<0.001).

The occurrence of first-ever stroke within the PCA territory was less common but still clearly appreciable (Figure 4B). The incidence of visuospatial neglect was high in patients with a right-hemispheric first-ever stroke in the PCA territory (52.38%) but was also not negligible in patients with a left-hemispheric first-ever stroke in the PCA territory (25%), with no significant differences in likelihood (right PCA: *P*=0.827; left PCA: *P*=0.083).

First-ever stroke within the anterior cerebral artery territory or within >1 territory was rare; hence, the data should be interpreted with caution. However, visuospatial neglect was observable also in some of these patients.

### Influence of the Assessment Type on Visuospatial Neglect Incidence After First-Ever Stroke Within the Cerebral Hemispheres

**Figure 5.**
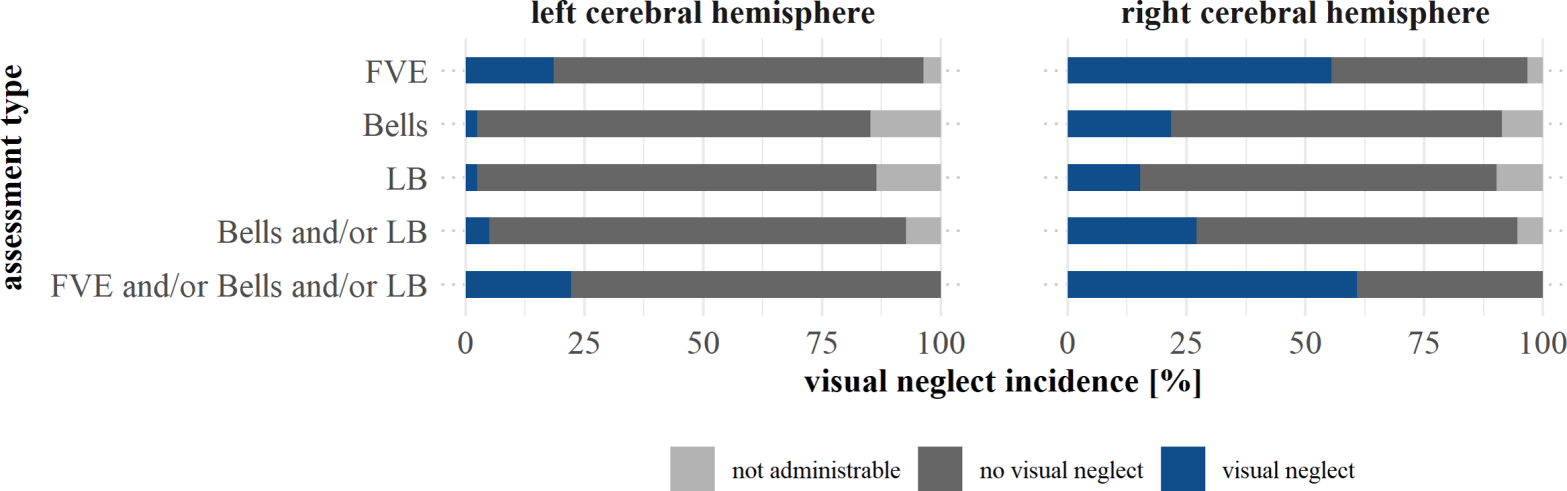
**Ability of the respective assessments and combinations thereof to detect visuospatial neglect.** Cumulative percentages (ie, every horizontal bar adds up to 100%) of cases in which contralesional visuospatial neglect was present or absent, or the administration of the respective assessment was not possible, for patients with first-ever stroke in the left (**left**) and right (**right**) cerebral hemisphere. Bells indicates Bells test;^13^ FVE, video oculography during free visual exploration; and LB, line bisection test.

FVE could be administered in 96.53% (167/173) of patients with first-ever stroke within the cerebral hemispheres and was, thus, significantly more feasible than the Bells test (administrable in 87.86% of patients, 152/173; *P*_corr_=0.006) and the line bisection test (administrable in 88.44% of patients, 153/173; *P*_corr_=0.008; Figure 5).

FVE detected visuospatial neglect significantly more often than the Bells test, the line bisection test, and a combination thereof. This was true for both patients with right-hemispheric (FVE versus Bells test: *P*_corr_<0.001; FVE versus line bisection test: *P*_corr_<0.001; and FVE versus Bells test+line bisection test: *P*_corr_<0.001) and left-hemispheric (FVE versus Bells test: *P*_corr_=0.016; FVE versus line bisection test: *P*_corr_=0.027; and FVE versus Bells test+line bisection test: *P*_corr_=0.026) cerebral first-ever strokes. Contrarily, a combination of the 3 assessments (FVE+Bells test+line bisection test) did not detect visuospatial neglect more often than FVE alone, both in patients with right-hemispheric (*P*_corr_=0.134) and left-hemispheric (*P*_corr_=0.480) cerebral first-ever strokes. Hence, administering the Bells test and the line bisection test additionally to FVE did not result in a significant advantage in terms of visuospatial neglect detection.

Subdividing the cases according to vascularization territories within the left and right cerebral hemispheres yielded similar results (Supplemental Results; Figure S2).

## Discussion

This single-center, prospective, observational cohort study identified, for the first time in an unselected and representative population, applying the most sensitive technique of FVE, an overall incidence (ie, irrespective of lesion location) of visuospatial neglect in the acute first-ever stroke of ≈38%. However, great differences were observable according to lesion location: visuospatial neglect incidence was ≈61% in acute right-hemispheric cerebral first-ever stroke and ≈22% in acute left-hemispheric cerebral first-ever stroke, in both hemispheres mainly concerning the MCA and PCA territory. Moreover, visuospatial neglect was less frequent but observable also after the first-ever stroke in less commonly affected locations, such as the left cerebellum, the pons, and the medulla oblongata, as well as multifocal first-ever stroke.

These figures are considerably higher than the ones identified by a recent systematic review^2^ considering newer epidemiological studies of visuospatial neglect (ie, after 2000), which reported an average incidence of ≈38% for acute right-hemispheric stroke (range, 19%–47%) and ≈17% for acute left-hemispheric stroke (range, 2%–24%). This discrepancy is consistent with the idea that cognitive impairment after stroke is still underrecognized^21^ and may be based on methodological differences.

First, several previous studies restricted the inclusion of patients according to first-ever stroke type or localization (eg, only ischemic, supratentorial, or anterior circulation).^2^ As shown, the incidence of visuospatial neglect after a first-ever stroke in the posterior circulation and atypical lesion sites (eg, cerebellar^22^ and infratentorial^23,24^) is not a negligible entity. We interpret the occurrence of visuospatial neglect after lesions to infratentorial structures as a consequence of the disconnection of cortico-ponto-cerebellar or tecto-cerebellar-tectal pathways (for details, see the study by Cazzoli et al^23^), connecting parietal, prefrontal, and temporal cortical areas to the cerebellum via the pons.^25^ This is consistent with the ever-increasing role of the cerebellum in cognitive and emotional processes (for a review, see the study by Schmahmann^26^). This is also in line with the perisylvian neural network theory of visuospatial neglect,^27^ as large parts of the cortico-ponto-cerebellar pathways seem to be temporo-ponto-cerebellar connections.^25^

Second, several previous studies excluded patients with aphasia, which, however, has an incidence of ≈30% after stroke,^28^ and determined the presence of visuospatial neglect with paper-pencil tests,^2^ which pose the problem of low sensitivity.^2,5^ As shown, FVE was significantly more feasible than paper-pencil tests, probably because this assessment is not hindered by the presence of aphasia^8^ (results concerning aphasia signs in our sample as assessed with the respective NIHSS item are reported in the Supplemental Results), motor impairment, and cognitive impairments other than visuospatial neglect, such as apraxia. This is relevant because the incidence of visuospatial neglect after a left-hemispheric cerebral first-ever stroke, one of the main causes of aphasia and apraxia, was considerable (≈22%) in the present study. Moreover, extending its application to the acute phase of stroke, FVE detected visuospatial neglect significantly more often than single paper-pencil tests and a combination thereof at the group level.^7–9^ As dissociations between different tests are always possible at the individual level, and as recommended by current international guidelines,^6^ the use of multiple tests is still advised whenever time and resources allow.

The relevance of these methodological differences can also be illustrated by the results of a recent, large investigation of the incidence of cognitive impairment after stroke. Gallucci et al^29^ studied 329 patients over 32 months, selecting only anterior circulation stroke and only nonaphasic individuals. In addition, the presence/absence of visuospatial neglect was determined with only 1 paper-pencil test. The relatively low reported incidence of visuospatial neglect (18.5%) seems, therefore, underestimated.

One may object that the observed high incidence of visuospatial neglect, particularly for the first-ever stroke affecting the MCA and PCA territory, would be due to visual field loss, a common sequela of first-ever stroke (incidence of ≈28%^30^). However, patients with stroke with visual field loss, but without visuospatial neglect, do not show any significant bias in the spatial distribution of visual fixations during visual exploration in the acute phase^31^ and may even show a contralesional bias in the subacute phase.^32^ This is clearly at odds with the criterion used to define the presence of visuospatial neglect in FVE in the present study, that is, a significant ipsilesional bias in the spatial distribution of visual fixations. Hence, it seems unlikely that our findings are influenced by the presence of a possible visual field loss (results concerning visual field loss as assessed with a confrontation method in our sample are reported in the Supplemental Results).

Failing to identify visuospatial neglect in acute stroke can be a missed opportunity with relevant consequences, given its strong, negative, and independent predictive power of poor outcomes, resulting in higher burdens and costs.^1^ For instance, an early identification (within 2 days after stroke) and, therefore, early treatment of visuospatial neglect can contribute to shorter hospital stays, that is, the difference in length of stay between patients with and without neglect can be eliminated.^33^

Our study also helped to identify several risk factors and stroke characteristics significantly associated with the incidence of visuospatial neglect in acute first-ever stroke. Patients with visuospatial neglect were older than their peers without visuospatial neglect. This result extends previous findings limited to acute right-hemispheric stroke.^34,35^ One possible explanation for this effect is the accumulation or increase in stroke risk factors with age, a typical example being atrial fibrillation.^36^ However, the association between age and incidence of visuospatial neglect persisted even after controlling for other significant factors (NIHSS score, atrial fibrillation, and thrombectomy), with each additional year of age being associated with ≈4% increase in the odds of having visuospatial neglect after first-ever stroke. Another hypothetical explanation, because the compensation of visuospatial neglect after stroke relies on specific patterns of spared structural connectivity,^37^ relies on the increased atrophy and white matter disease often observed in elderly patients.^38^ Moreover, patients with visuospatial neglect had a higher NIHSS, were more likely to receive thrombectomy, and had more commonly atrial fibrillation than their peers without visuospatial neglect. A higher NIHSS in patients with visuospatial neglect is consistent with the previously described higher severity of stroke at admission.^35,38^ The association between the presence of visuospatial neglect and receiving thrombectomy is probably linked to the latter being most established in large vessel occlusions within the anterior circulation.^39^ This type of first-ever stroke is far more common, generally, and in our patient sample, and has a higher incidence of visuospatial neglect. In addition, virtually all field tests for the identification of large vessel occlusion, and thus candidates for thrombectomy, include an item concerning gaze deviation,^40^ which is, in turn, strongly associated with visuospatial neglect early poststroke.^41^ The association between visuospatial neglect and atrial fibrillation is consistent with the fact that patients with atrial fibrillation have a higher risk of cognitive impairment and worse outcomes after stroke, with hypothesized mechanisms being larger stroke volume, more severe hypoperfusion, and higher risk of hemorrhagic transformation.^36^ This, together with the fact that an undetermined cause in the TOAST classification includes cases with >1 possible cause, is also a hypothetical explanation as to why patients with visuospatial neglect were less likely to present with an undetermined cause.

Our study has also some limitations. First, although data collection was conducted in a stroke center with a large, both urban and rural, catchment area, the study was single center. Future multicentric studies could confirm the findings’ validity across multiple institutions in contexts other than Central Europe. Second, we considered only visuospatial egocentric neglect, which is often seen as the most common form of neglect.^42^ Further consideration of less commonly assessed forms of neglect may have led to even higher incidences; for instance, allocentric neglect has been shown to be more common than egocentric neglect after left-hemispheric stroke.^43^ In addition, our study used a version of the classic line bisection test, in which patients are asked to bisect multiple lines on the same sheet of paper and the deviation between the actual and marked midlines is scored. Novel approaches, which propose to present the lines to be bisected singularly and to apply an alternative assessment using an end point weighting bias, have been shown to identify neglect more reliably than the above standard measures of line bisection deviation.^44^ These aspects should be considered in future studies. Third, our study provides some insight into the incidence of visuospatial neglect according to macroscopic brain segments and vascularization territories within the cerebral hemispheres only. Future studies should additionally assess the influence of more advanced imaging parameters, such as hypoperfusion, which can be estimated using common magnetic resonance imaging sequences such as fluid-attenuated inversion recovery sequences (eg, the study by Bunker et al^45^) and lesion volume (eg, the study by Sperber^46^). Fourth, the video oculography apparatus may be considered specialist equipment, somewhat limiting its broader application.^6^ However, recent technological developments show the feasibility of eye-tracking by means of omnipresent devices such as tablets or smartphones.^47^ This should soon allow to apply adapted FVE paradigms without any specialist equipment.

In conclusion, the incidence of visuospatial neglect in an unselected cohort, using a highly sensitive assessment, is considerably higher than previously assumed and can also occur after less typically localized strokes. These results can enhance the awareness of visuospatial neglect in the acute setting, potentially facilitating earlier identification and therapy of this disabling disorder.

## Article Information

### Acknowledgments

The authors are grateful to the patients who participated in our study and to the clinical team at the Neurocenter of the Kantonsspital Luzern for their assistance and support.

### Sources of Funding

This work was supported by the Swiss Heart Foundation grant FF21037 to Dr Cazzoli and the Swiss National Science Foundation grants 320030_169789 and 488 32003b_196915 to Dr Nyffeler.

### Disclosures

None.

### Supplemental Material

Supplemental Methods

Supplemental Results

Figures S1–S2

References 7, 20, 48–50
